# Detection Rate of Culprit Tumors Causing Osteomalacia Using Somatostatin Receptor PET/CT: Systematic Review and Meta-Analysis

**DOI:** 10.3390/diagnostics10010002

**Published:** 2019-12-18

**Authors:** Marie Meyer, Marie Nicod Lalonde, Nathalie Testart, Mario Jreige, Christel Kamani, Sarah Boughdad, Barbara Muoio, Fabio Becce, Niklaus Schaefer, Christian Candrian, Luca Giovanella, John O. Prior, Giorgio Treglia, Martin Riegger

**Affiliations:** 1 Department of Nuclear Medicine and Molecular Imaging, Lausanne University Hospital, CH-1011 Lausanne, Switzerland; Marie-Madeleine.Meyer@chuv.ch (M.M.); Marie.Nicod-Lalonde@chuv.ch (M.N.L.); Nathalie.Testart@chuv.ch (N.T.); Mario.Jreige@chuv.ch (M.J.); Christel-Hermann.Kamani@chuv.ch (C.K.); Sarah.Boughdad@chuv.ch (S.B.); Niklaus.Schaefer@chuv.ch (N.S.); John.Prior@chuv.ch (J.O.P.); 2 Clinic of Medical Oncology, Oncology Institute of Southern Switzerland, Ente Ospedaliero Cantonale, CH-6500 Bellinzona, Switzerland; Barbara.Muoio@eoc.ch; 3 Department of Diagnostic and Interventional Radiology, Lausanne University Hospital, CH-1011 Lausanne, Switzerland; Fabio.Becce@chuv.ch; 4 Faculty of Biology and Medicine, University of Lausanne, CH-1005 Lausanne, Switzerland; 5 Clinic of Orthopaedics and Traumatology, Department of Surgery, Ospedale Regionale di Lugano, Ente Ospedaliero Cantonale, CH-6900 Lugano, Switzerland; Christian.Candrian@eoc.ch; 6 Clinic of Nuclear Medicine, Imaging Institute of Southern Switzerland, Ente Ospedaliero Cantonale, CH-6500 Bellinzona, Switzerland; Luca.Giovanella@eoc.ch; 7 Faculty of Medicine, University of Zurich, CH-8091 Zurich, Switzerland; 8 Health Technology Assessment Unit, Academic Education Research and Innovation Area, General Directorate, Ente Ospedaliero Cantonale, CH-6500 Bellinzona, Switzerland; 9 Clinic of Orthopedics and Traumatology, Department of Surgery, Ospedale Regionale di Bellinzona, Ente Ospedaliero Cantonale, CH-6500 Bellinzona, Switzerland; Martin.Riegger@eoc.ch

**Keywords:** PET, osteomalacia, culprit tumor, somatostatin, detection rate, systematic review, meta-analysis

## Abstract

Background: Tumor-induced or oncogenic osteomalacia (TIO) is a rare paraneoplastic syndrome in which osteomalacia is a consequence of fibroblast growth factor 23 (FGF23) secretion by a mesenchymal tumor. The localization of the culprit lesion in patients with TIO is often challenging. Several studies have evaluated the detection rate (DR) of these tumors using somatostatin receptor positron emission tomography (SSTR-PET/CT). We aimed to summarize literature findings on this topic providing pooled estimates of DR. Methods: A comprehensive literature search by screening PubMed, Embase and Cochrane library electronic databases through August 2019 was performed. The pooled DR of culprit tumors using SSTR-PET/CT in patients with TIO was calculated using a random-effects statistical model. Results: Fourteen studies on the use of SSTR-PET/CT in detecting the culprit tumor in patients with TIO were included in the qualitative analysis. The pooled DR of SSTR-PET/CT on a per-patient-based analysis calculated using eleven studies (166 patients) was 87.6% (95% confidence interval (95% CI) 80.2–95.1%). Statistical heterogeneity among studies was detected (I-square = 63%), likely due to the use of different radiolabeled somatostatin analogues, as demonstrated by a subgroup analysis. Conclusions: Despite limited literature data due to the rarity of the disease, SSTR-PET/CT demonstrated a very high DR of culprit tumors in patients with TIO and it could be used as first-line imaging method for this indication.

## 1. Introduction

Tumor-induced or oncogenic osteomalacia (TIO) is a rare paraneoplastic syndrome [1,2]. The majority of TIO cases are caused by phosphaturic mesenchymal tumors [3]. The culprit tumors of TIO produce fibroblast growth factor 23 (FGF23), a protein which regulates renal phosphate handling and 25-hydroxyvitamin D 1α-hydroxylase activity. The hypersecretion of FDG-23 may cause hypophosphatemia due to a decreased tubular phosphate reabsorption and a low level of active vitamin D. Chronic hypophosphatemia could eventually lead to inadequate bone mineralization, presenting as osteomalacia [1,2].

On a clinical point of view, the manifestation of TIO is mainly musculoskeletal, such as bone pain, fractures and muscle weakness. Due to its nonspecific clinical presentation or lack of awareness, the diagnosis of TIO is often significantly delayed, resulting in physical suffering or psychological distress for the patients. The diagnosis of TIO should be considered in patients with hypophosphatemia and osteomalacia—or rickets—but a differential diagnosis from other disorders of phosphate metabolism should be undertaken [1,2].

The accurate and early detection and localization of culprit tumors in patients with TIO is crucial for patient management and treatment. The successful detection and complete surgical resection of the culprit tumors typically leads to the rapid resolution of symptoms or the reversal of biochemical imbalance [1]. The detection of culprit tumors in patients with TIO may be challenging, since the majority of these tumors are very small and can be localized everywhere in the body. A combination of thorough physical examination, laboratory tests and imaging techniques should be applied for the diagnosis of TIO and the localization of culprit tumors [1].

Regarding imaging methods, several techniques can be used for the localization of culprit tumors in patients with TIO, including anatomic imaging modalities such as plain radiographs, computed tomography (CT), and magnetic resonance imaging (MRI), nuclear medicine imaging techniques or hybrid functional and morphological imaging modalities such as single-photon emission computed tomography/CT (SPECT/CT), or positron emission tomography/CT (PET/CT) using different radiopharmaceuticals [4].

Recent literature data have demonstrated that somatostatin receptor PET/CT (SSTR-PET/CT), using different somatostatin analogues (e.g., DOTANOC, DOTATATE, DOTATOC) labelled with Gallium-68 (^68^Ga), may have a promising role as an imaging modality in the detection and localization of culprit tumors in patients with TIO [5]. SSTR-PET/CT is extensively used for the diagnosis of neuroendocrine tumors, due to the overexpression of SSTRs in neuroendocrine tumor cells [6,7,8]. However, SSTRs are also expressed in non-neuroendocrine tumors [9], such as mesenchymal tumors [4,5]; therefore, as most of culprit lesions in patients with TIO are mesenchymal tumors, they can be detected using SSTR-PET/CT [4,5].

We aimed to perform a systematic review and meta-analysis on the detection rate (DR) of culprit tumors in patients with TIO using SSTR-PET/CT to provide evidence-based data that could be useful in justifying the use of this technique as first-line imaging method for this indication.

## 2. Methods

This systematic review and the related meta-analysis were written according to the “Preferred Reporting Items for a Systematic Review and Meta-Analysis of Diagnostic Test Accuracy Studies” (PRISMA-DTA statement), a guideline which describes the items required for reporting in systematic reviews and meta-analyses of DTA studies [10]. Furthermore, specific suggestions for systematic reviews of diagnostic imaging studies were followed [11,12].

### 2.1. Search Strategy

Three co-authors (M.M, M.N.L. and G.T.) independently performed a comprehensive computer literature search of the PubMed/MEDLINE, Cochrane library and Embase databases to find relevant published articles on the DR of culprit tumors in patients with TIO using SSTR-PET/CT.

This search string—based on a combination of key words, Boolean operators and truncations (*) —was created and used: (A) “DOTA*’ OR ‘somatostatin’ AND (B) ‘PET’ OR ‘positron*’ AND (C) ‘osteomalacia’ OR ‘culprit’ OR ‘mesench*’. No beginning date limit nor language restrictions were used. The literature search was updated until 31st August 2019. To expand the literature search, the references of the retrieved articles were also screened for possible additional records.

### 2.2. Study Selection

Studies assessing the DR of culprit tumors in patients with TIO using SSTR-PET/CT were eligible for inclusion in the qualitative analysis (systematic review).

The exclusion criteria for the systematic review were: (a) articles not within the field of interest; (b) reviews, editorials, letters, comments, conference proceedings; (c) case reports and small case series (less than 5 patients included).

All the studies included in the systematic review were included in the meta-analysis, except those with possible patient data overlap. If studies with possible patient data overlap were found, only the article with more complete information was included in the meta-analysis.

Three co-authors (M.M, M.N.L. and G.T.) independently screened the abstracts of the retrieved articles, applying the predefined inclusion and exclusion criteria. Subsequently, the researchers independently reviewed the full text of the selected articles to assess their eligibility for inclusion in the systematic review. Any disagreement was solved through a consensus meeting among the researchers performed in September 2019 at the Department of Nuclear Medicine and Molecular Imaging of the University Hospital of Lausanne, Switzerland.

### 2.3. Data Extraction

For each selected article, information was collected on basic study characteristics (authors, year of publication, country, study design), patient characteristics (type and number of patients evaluated, age and sex ratio, FGF23 serum levels), technical details (type of hybrid imaging used, radiolabeled somatostatin analogues used, injected activity, time between radiopharmaceutical injection and PET/CT image acquisition, image analysis and other functional imaging methods performed for comparison), data on DR, and the site of the culprit tumors detected by SSTR-PET/CT, including the number and type of tumors proven by histopathology.

### 2.4. Quality Assessment

The quality of the studies included in this systematic review was critically appraised using the revised “Quality Assessment of Diagnostic Accuracy Studies” tool (QUADAS-2) [13].

### 2.5. Statistical Analysis

The DR of culprit tumors using SSTR-PET/CT was obtained from individual studies on a per-patient-based analysis. A random-effects model was used for the statistical pooling of DR. Pooled data were presented with 95% confidence intervals (95% CI) and displayed using forest plots. Heterogeneity was estimated using the I-square index (I^2^); a statistical heterogeneity among studies was present if I^2^ was higher than 50% [14]. If significant heterogeneity was found, subgroup analyses taking into account the type of radiolabeled somatostatin analogues used (DOTATATE, DOTATOC, DOTANOC) were performed. Publication bias was assessed through the visual evaluation of a funnel plot and the Egger’s test [15].

Statistical analyses were performed using OpenMeta[Analyst]^®^ software (version 0.1503, Agency for Healthcare Research and Quality, Rockville, MD, USA) and StatsDirect software version 3 (StatsDirect Ltd., Cambridge, UK).

## 3. Results

### 3.1. Literature Search

**Figure 1:**
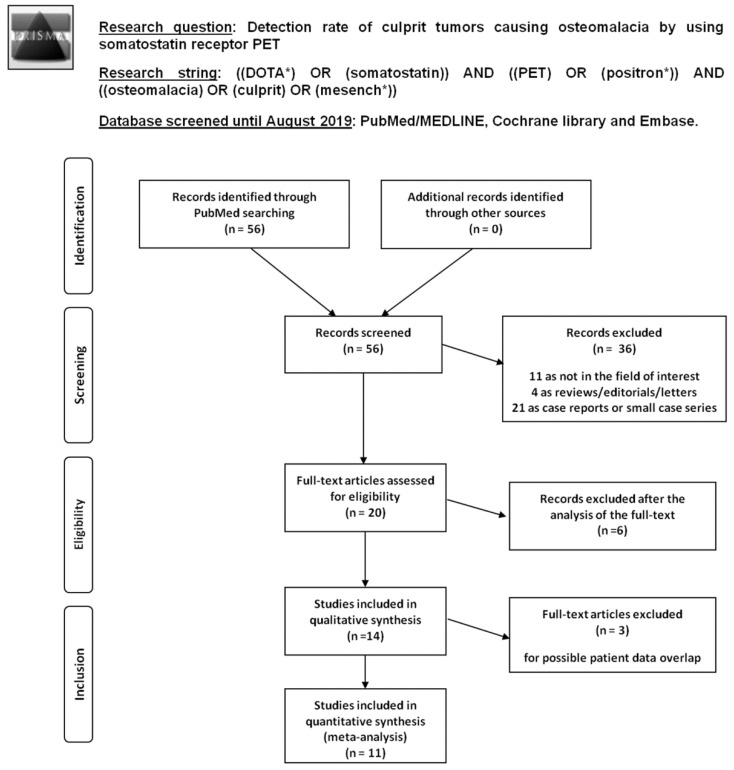
Flow chart of the search for eligible studies on the detection rate of culprit tumors causing osteomalacia using somatostatin receptor positron emission tomography (SSTR-PET/CT).

Literature search results are summarized in Figure 1 and briefly described below.

**Figure 2:**
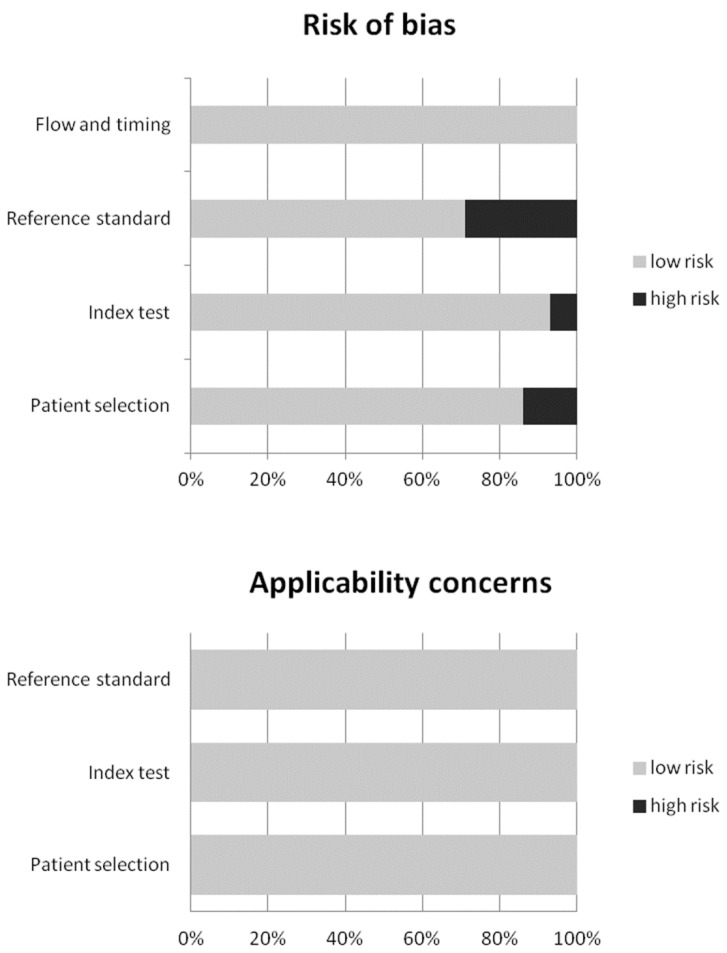
Overall quality assessment of the studies included in the systematic review according to the Quality Assessment of Diagnostic Accuracy Studies (QUADAS-2) tool.

Overall, 56 records were identified through the comprehensive computer literature search of the PubMed/MEDLINE, Cochrane library and Embase databases. Screening 56 abstracts, 36 records were excluded: 11 as they were not in the field of interest, four as reviews/editorials/letters, 21 as case reports or small case series (less than five patients included). Twenty articles were selected and their full text was retrieved. No additional records were found screening the references of these articles, whereas six articles were excluded after the analysis of the full text. Therefore, 14 articles were included in the qualitative analysis (systematic review) [16,17,18,19,20,21,22,23,24,25,26,27,28,29]. Three articles were excluded from the meta-analysis for possible patient data overlap [18,25,26]. Overall, 11 articles (166 patients with TIO) were included in the quantitative analysis (meta-analysis) [16,17,19,20,21,22,23,24,27,28,29]. The characteristics of the 14 studies included in the systematic review are presented in Table 1 and Table 2. The diagnostic accuracy data from these articles are shown in Table 3. The quality appraisal of studies included in the systematic review is reported in Figure 2.

### 3.2. Qualitative Analysis (Systematic Review)

#### 3.2.1. Basic Study and Patient Characteristics

Screening the selected databases, 14 articles evaluating the DR of culprit tumors in patients with TIO using SSTR-PET/CT were selected (Table 1) [16,17,18,19,20,21,22,23,24,25,26,27,28,29]. Most of the selected articles were retrospective (93%) or single-center (86%) studies. All the selected articles were published in the last 6 years (from 2013 to 2019) by research groups of different continents (Asia, Europe, America and Oceania), but studies from Asia were the most represented (71%). The patients included in the selected articles have a clinical and biochemical diagnosis of TIO or a suspected TIO. The mean age of the patients included in these studies ranged from 36 to 53 years and the percentage of male patients (sex ratio) largely ranged from 17% to 80%. The majority of patients included in the selected studies had symptomatic osteomalacia with hypophosphatemia and evidence of increased serum levels of FGF23. The most common presenting symptoms were bone pain and muscle weakness [16,17,18,19,20,21,22,23,24,25,26,27,28,29].

#### 3.2.2. Technical Aspects

Technical details about SSTR-PET/CT from the included studies are summarized in Table 2. Hybrid PET/CT was performed in 100% of the studies, without contrast-enhanced CT in the majority of cases (93%). The injected radiolabeled somatostatin analogues were ^68^Ga-DOTATATE (in 10 studies), ^68^Ga-DOTANOC (in two studies) and ^68^Ga-DOTATOC (in one study). The remaining study used both ^68^Ga-DOTATATE and ^68^Ga-DOTANOC. The radiopharmaceutical injected activity was quite different among the studies. The time interval between radiopharmaceutical injection and SSTR-PET/CT acquisition ranged from 20 min to 90 min. A whole-body PET/CT acquisition (from head to toes) was performed in all the studies. The analysis of SSTR-PET/CT images was performed using qualitative criteria (visual analysis) in all the studies and additional semi-quantitative parameters, as the maximal standardized uptake value (SUV_max_), in 64% of cases. At visual analysis, the areas of focal increased radiopharmaceutical uptake greater than the surrounding tissue and not judged as physiological activity were considered abnormal.

Other functional imaging modalities were used for the comparison of SSTR-PET/CT findings in most of the articles; in particular SSTR scintigraphy or SPECT/CT (using ^111^In- or ^99m^Tc-octreotide), fluorine-18 fluorodeoxyglucose (^18^F-FDG) PET/CT, ^99m^Tc-sestamibi scintigraphy and bone scintigraphy.

Histopathological results (gold standard) and/or clinical/imaging/biochemical follow-up were used as the reference standard in the included studies.

#### 3.2.3. Main Findings

As shown in Table 3, most of the selected studies showed a good DR of culprit tumors in patients with TIO using SSTR-PET/CT. The culprit lesions were usually small benign tumors located in the bones or soft tissues, presenting a high uptake of radiolabeled somatostatin analogues (^68^Ga-DOTATATE, ^68^Ga-DOTATOC or ^68^Ga-DOTANOC) at SSTR-PET/CT [16,17,18,19,20,21,22,23,24,25,26,27,28,29]. The most frequent site of culprit tumors were the lower limbs; other frequent sites were the cranio-facial region and the trunk, whereas the localization of culprit tumors in the upper limbs was less frequent. Most of the culprit tumors detected by SSTR-PET/CT were confirmed by histopathology and phosphaturic mesenchymal tumor was the most frequent histological type. Malignant or metastatic tumors detected by SSTR-PET/CT in patients with TIO were rare [16,17,18,19,20,21,22,23,24,25,26,27,28,29]. A very high inter-observer concordance among PET/CT masked readers was reported by one study for the visual detection of culprit tumors by SSTR-PET/CT [20]. Interestingly, a significant correlation between SUVmax of the culprit tumor at SSTR-PET/CT and serum FGF23 levels was not reported [17,20]. Furthermore, there was no significant difference for any of the biochemical parameters and for the duration of the disease between SSTR-PET/CT-positive and SSTR-PET/CT-negative cases [20].

For the majority of patients with TIO included in the selected studies, before performing SSTR-PET/CT, conventional imaging (including CT and MRI) failed to detect the culprit tumors. This has delayed the recognition of TIO and/or led to difficulties in localizing the culprit tumor once TIO was suspected [16,17,18,19,20,21,22,23,24,25,26,27,28,29]. When a culprit tumor was detected by SSTR-PET/CT, anatomical localization studies using additional CT or MRI were performed in some patients, for the localization and characterization of the lesion where the surgeon deemed it necessary for surgical intervention. Notably, in some cases, the area of functional abnormality detected by SSTR-PET did not correspond to any morphological change on CT or MRI [16,17,18,19,20,21,22,23,24,25,26,27,28,29].

Fractures can be a common consequence of TIO, and they can also lead to increased radiopharmaceutical uptake at SSTR-PET/CT, potentially affecting the accuracy of this method in detecting the culprit tumors. However, it has been demonstrated that mild radiopharmaceutical uptake at the sites of fracture is not a major challenging factor in the interpretation of SSTR-PET/CT when both the intensity of the radiopharmaceutical uptake at PET and the morphology of CT are assessed. In fact, fractures show a characteristic morphology at CT, and they usually present a lower radiopharmaceutical uptake at SSTR-PET compared to culprit tumors in patients with TIO [19,21]. Inflammatory lesions, such as granulomatous lesions, may also cause false positive findings for culprit tumors in patients with TIO, due to the high expression of SSTRs by activated inflammatory cells [20,25].

When compared to ^18^F-FDG PET/CT, SSTR-PET/CT had a higher DR of culprit tumors in patients with TIO. Additionally, ^18^F-FDG PET/CT may lead to a higher number of false positive results compared to SSTR-PET/CT for this indication. Furthermore, in those TIO patients with culprit tumors positive at both ^18^F-FDG PET/CT and SSTR-PET/CT, the lesion-to-background contrast was higher at SSTR-PET/CT compared to that of ^18^F-FDG PET/CT, enabling a more confident diagnosis [16,17,20,22,23,26,28,29].

Compared to SSTR scintigraphy and SPECT/CT, SSTR-PET/CT showed a higher DR of culprit tumors in patients with TIO [17,18,20,23,27,28,29].

Bone scintigraphy was performed in some studies, but it showed a significantly lower DR of the culprit tumor in patients with TIO compared to SSTR-PET/CT. Furthermore, bone scintigraphy may show areas of focal radiopharmaceutical uptake at the site of fractures or scintigraphic signs suggestive of metabolic bone disease in patients with TIO [16,24,29].

Overall, allowing the detection of culprit tumors which remained occult after conventional work-up, SSTR-PET/CT induced a change of management in a significant percentage of patients with TIO. In particular, in most cases of culprit tumors detected by SSTR-PET/CT, patients with TIO were referred to surgery for the excision of the culprit tumor. The most frequent outcome after surgery was normalization of biochemical parameters and clinical remission of TIO [16,17,18,19,20,21,22,23,24,25,26,27,28,29].

### 3.3. Quantitative Analysis (Meta-Analysis)

**Figure 3:**
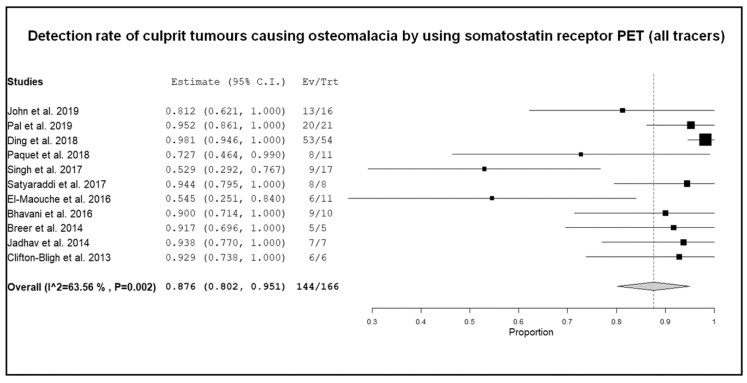
Forest plot of individual studies and pooled detection rate of culprit tumors causing osteomalacia using SSTR-PET/CT, including 95% confidence intervals. The size of the squares indicates the weight of each study.

**Figure 4:**
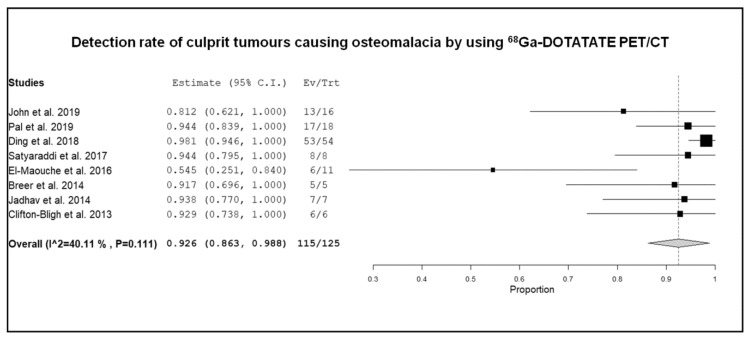
Forest plot of individual studies and pooled detection rate of culprit tumors causing osteomalacia using 68Ga-DOTATATE PET/CT, including 95% confidence intervals. The size of the squares indicates the weight of each study.

**Figure 5:**
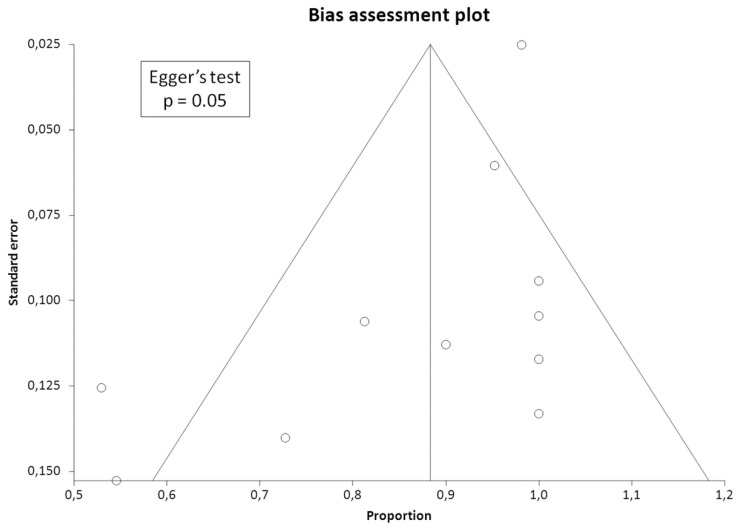
Funnel plot of the included studies about the detection rate of culprit tumors causing osteomalacia using SSTR-PET/CT. The plot demonstrates an asymmetric distribution of the outcome measure suggesting the presence of a possible bias. The little circles in the images represent the outcome measure of the single studies.

Eleven studies including 166 patients with TIO were selected for the meta-analysis [16,17,19,20,21,22,23,24,27,28,29]. Results of the meta-analysis are shown in Figure 3, Figure 4 and Figure 5.

The DR of culprit tumors using SSTR-PET/CT in patients with TIO ranged from 53% to 100%, with a pooled outcome measure of 87.6% (95% CI: 80.2–95.1%) (Figure 3). A moderate statistical heterogeneity among the included studies was found (I^2^ = 63.5%).

To explore the statistical heterogeneity, a subgroup analysis was performed taking into account the different type of radiolabeled somatostatin analogues used for SSTR-PET/CT (^68^Ga-DOTATATE, ^68^Ga-DOTANOC, ^68^Ga-DOTATOC), but a significant number of studies was available only for ^68^Ga-DOTATATE (*n* = 8), whereas a paucity of studies was available for the other radiopharmaceuticals (^68^Ga-DOTATOC and ^68^Ga-DOTANOC). As shown by Figure 4, the pooled DR of culprit tumors causing osteomalacia using ^68^Ga-DOTATATE was 92.6% (95% CI: 86.3–98.8%), but in this subgroup analysis a significant statistical heterogeneity was not detected (I^2^ < 50%). Overall, the DR of culprit tumor was similar using the different radiolabeled somatostatin analogues [16,17,18,19,20,21,22,23,24,25,26,27,28,29].

A funnel plot was created to evaluate the publication bias. An asymmetry was evident at funnel plot, thus demonstrating the presence of bias (Figure 5). The publication bias was also confirmed by the result of the Egger’s test (*p* = 0.05).

## 4. Discussion

To the best of our knowledge, this is the first systematic review and meta-analysis which has evaluated the DR of culprit tumors in patients with TIO using SSTR-PET/CT. Several studies have been published on this topic, but these studies have a limited statistical power, as a small number of patients with TIO were enrolled due to the rarity of the disease. Therefore, we have pooled data reported in the published studies, to obtain more robust estimates on the DR of SSTR-PET/CT in this setting.

Our systematic review and meta-analysis demonstrated a very good DR of culprit tumors in patients with TIO using SSTR-PET/CT—with a pooled value of about 90%—due to the overexpression of SSTRs in most of the culprit tumors causing osteomalacia. Even if false negative findings are possible, in about 10% of cases, it should be underlined that SSTR-PET/CT has allowed the detection of culprit tumors which remained occult with conventional imaging methods in most of the cases [16,17,18,19,20,21,22,23,24,25,26,27,28,29]. The main explanation for false negative results at conventional imaging methods is the reduced size of most culprit tumors causing osteomalacia [1,3,4]. False positive findings of SSTR-PET/CT in this setting are also described [20,25], in particular caused by inflammatory lesions due to the overexpression of SSTR by activated inflammatory cells [30,31]. Granulomatous lesions may be positive at SSTR-PET/CT in some cases and it could be difficult to differentiate them from tumors causing osteomalacia—or neuroendocrine tumors—using conventional imaging methods or SSTR-PET/CT. As culprit tumors detected by SSTR-PET/CT may be located everywhere throughout the body [16,17,18,19,20,21,22,23,24,25,26,27,28,29], it is important to perform a whole-body SSTR-PET/CT acquisition (from head to toes) to avoid missing lesions.

Regarding the comparison with other functional and hybrid imaging techniques, SSTR-PET/CT resulted as being clearly superior to bone scintigraphy, SSTR scintigraphy and SPECT/CT, and ^18^F-FDG-PET/CT in terms of DR of culprit tumors in patients with TIO [16,17,18,20,22,23,24,26,27,28,29]. Beyond the superior DR, SSTR-PET/CT has an even higher specificity compared to bone scintigraphy and ^18^F-FDG-PET/CT, due to the higher number of false positive findings that can be obtained using the latter imaging methods for this indication [16,17,20,22,23,24,26,28,29]. The higher DR of SSTR-PET/CT compared to SSTR scintigraphy or SPECT/CT mainly derives from the superior spatial resolution of PET/CT compared to planar imaging and SPECT/CT [17,18,20,23,27,28,29].

Semi-quantitative PET analysis (e.g., using SUVmax) can be used as an adjunct tool to visual PET analysis for SSTR-PET/CT interpretation, in particular to make a differential diagnosis between culprit tumors in patients with TIO and TIO-related fractures [19,21].

Contrast enhancement could further improve the DR of culprit tumors using SSTR-PET/CT in patients with TIO [16], but data on this regard are very limited and this should be better evaluated in further studies. Another topic that would need to be evaluated in further studies is the diagnostic performance of SSTR-PET/MRI compared to SSTR-PET/CT in this setting [32], because data on this regard are still lacking.

Nowadays, evidence-based data are crucial to establish the PET/CT indications that should be covered by medical insurances worldwide [33]. Overall, our systematic review and meta-analysis provides evidence-based data which could support the use of SSTR-PET/CT as first-line imaging method in detecting culprit tumors in patients with TIO. Beyond the DR, SSTR-PET/CT induced a change of management in a significant percentage of patients with TIO [16,17,18,19,20,21,22,23,24,25,26,27,28,29]. In particular, in most cases of culprit tumors detected by SSTR-PET/CT, patients with TIO were referred to surgery with subsequent normalization of biochemical parameters and the clinical remission of TIO in the majority of cases [16,17,18,19,20,21,22,23,24,25,26,27,28,29]. Further prospective and multicenter studies—and, in particular, cost-effectiveness analyses—could strengthen the role of SSTR-PET/CT in this setting.

Some limitations and biases of our systematic review and meta-analysis should be considered. First of all, a quite limited number of studies and patients were available for the systematic review and the meta-analysis, but this is justified by the rarity of TIO. Moreover, as a composite reference standard was used in some studies, a possible verification bias could not be excluded in some cases; nevertheless, most of the culprit tumors detected by SSTR-PET/CT were verified by histopathology. Heterogeneity among studies (i.e., due to differences in patient characteristics, methodological aspects and study quality) may represent a bias in a meta-analysis. We have detected a statistical heterogeneity among the included studies in our meta-analysis, but we have explored this heterogeneity performing a subgroup analysis based on the radiopharmaceutical used for SSTR-PET/CT, thus demonstrating that the different PET radiopharmaceutical used may be cause of moderate heterogeneity even if the DR of culprit tumors were similar using different radiopharmaceuticals for SSTR-PET/CT. Lastly, we found a publication bias as demonstrated by the funnel plot and the Egger’s test; therefore, the outcome of the studies has influenced the decision whether to publish or not the articles. We have tried to limit the publication bias, excluding from the analysis those studies including less than five patients with TIO.

## 5. Conclusions

Despite limited literature data due to the rarity of the disease, SSTR-PET/CT demonstrated a very high DR of culprit tumors in patients with TIO, and it could be used as first-line imaging method for this indication. Further prospective and multicenter studies, and in particular cost-effectiveness analyses, could strengthen the role of SSTR-PET/CT in this setting.

## Figures and Tables

**Table 1:** Basic study and patient characteristics of the included studies.

Authors	Year	Country	Study Design	Type of Patients Evaluated	Number of Patients with TIO Referred for SSTR-PET/CT	Age (years)	%Male	FGF23 Serum Level
John et al. [16]	2019	India	Retrospective single centre	Patients with clinical and biochemical diagnosis of TIO	16	Mean: 45 (18–61)	75%	112–1500 RU/mL
Pal et al. [17]	2019	India	Retrospective multicentre	Patients with clinical and biochemical diagnosis of TIO	21	Mean: 40.2 (19–58)	38%	102–6435 RU/mL
Zhang et al. [18]	2018	China	Retrospective single centre	Patients with clinical and biochemical diagnosis of TIO and negative ^99m^Tc-octreotide SPECT	37	Mean: 44 (17–75)	59%	NR
Ding et al. [19]	2018	China	Retrospective single centre	Patients with clinical and biochemical diagnosis of TIO	54	Mean: 41.2 (15–82)	63%	NR
Paquet et al. [20]	2018	France	Retrospective single centre	Patients with clinical and biochemical diagnosis of TIO	15	Mean: 53 (23–83)	67%	29–1916 RU/mL
Singh et al. [21]	2017	India	Retrospective single centre	Patients with suspected TIO	17	Mean: 42.4 (18–70)	47%	59–12000 RU/mL
Satyaraddi et al. [22]	2017	India	Retrospective single centre	Patients with clinical and biochemical diagnosis of TIO	8	Mean: 46.6 (18–74)	50%	202–3556 RU/mL
El-Maouche et al. [23]	2016	USA	Prospective single centre	Patients with clinical and biochemical diagnosis of TIO	11	Mean: 38 (19–60)	45%	105–5939 pg/mL
Bhavani et al. [24]	2016	India	Retrospective single centre	Patients with clinical and biochemical diagnosis of TIO	10	Mean: 40 (13–53)	80%	152–2323 RU/mL
Zhang et al. [25]	2015	China	Retrospective single centre	Patients with suspected TIO	54	Mean: 42.2 (19–68)	48%	NR
Agrawal et al. [26]	2015	India	Retrospective single centre	Patients with suspected TIO	6	Mean: 37.5 (26–55)	17%	148–6685 RU/mL
Breer et al. [27]	2014	Germany	Retrospective single centre	Patients with suspected TIO	5	Mean: 50.2 (41–62)	40%	<9.9–78.3 pg/nL
Jadhav et al. [28]	2014	India	Retrospective single centre	Patients with clinical and biochemical diagnosis of TIO	7	Mean: 35.7 (22–49)	71%	109–6000 RU/mL
Clifton-Bligh et al. [29]	2013	Australia	Retrospective multicentre	Patients with clinical and biochemical diagnosis of TIO	6	Mean: 43.5 (28–65)	50%	59–1940 ng/L

Legend: FGF23 = Fibroblast Growth Factor-23; TIO = Tumor-Induced Osteomalacia; PET/CT = Positron Emission Tomography/Computed Tomography; SPECT = Single Photon Emission Computed Tomography; SSTR = somatostatin receptor.

**Table 2:** Technical aspects of the included studies.

Authors	Hybrid Imaging Modality	Tracer Used	Injected Activity	Time Interval between Radiotracer Injection and Image Acquisition	Image Analysis	Other Functional Imaging Modalities Performed for Comparison
John et al. [16]	PET/CT (contrast enhanced CT)	^68^Ga-DOTATATE	75–185 MBq	30–45 min	Visual	bone scintigraphy ^18^F-FDG PET/CT
Pal et al. [17]	PET/CT (low-dose CT)	^68^Ga-DOTATATE, ^68^Ga-DOTANOC	NR	NR	Visual and semi-quantitative (SUVmax)	^99m^Tc-octreotide SPECT/CT ^18^F-FDG PET/CT
Zhang et al. [18]	PET/CT (low-dose CT)	^68^Ga-DOTATATE	44–111 MBq	40–60 min	Visual and semi-quantitative (SUVmax)	^99m^Tc-octreotide SPECT/CT
Ding et al. [19]	PET/CT (low-dose CT)	^68^Ga-DOTATATE	NR	NR	Visual and semi-quantitative (SUVmax)	
Paquet et al. [20]	PET/CT (low-dose CT)	^68^Ga-DOTATOC	1.6 MBq/kg	60 min	Visual and semi-quantitative (SUVmax, BTV)	^111^In-octreotide SPECT/CT ^18^F-FDG PET/CT
Singh et al. [21]	PET/CT (low-dose CT)	^68^Ga-DOTANOC	111–148 MBq	45 ± 15 min	Visual and semi-quantitative (SUVmax)	
Satyaraddi et al. [22]	PET/CT (low-dose CT)	^68^Ga-DOTATATE	NR	NR	Visual	^18^F-FDG PET/CT ^99m^Tc-red blood cells scintigraphy
El-Maouche et al. [23]	PET/CT (low-dose CT)	^68^Ga-DOTATATE	185 MBq	60 min	Visual and semi-quantitative (SUVmax)	^111^In-octreotide SPECT/CT ^18^F-FDG PET/CT
Bhavani et al. [24]	PET/CT (low-dose CT)	^68^Ga-DOTANOC	111–185 MBq	60 min	Visual and semi-quantitative (SUVmax)	bone scintigraphy ^99m^Tc-sestamibi scintigraphy
Zhang et al. [25]	PET/CT (low-dose CT)	^68^Ga-DOTATATE	111–148 MBq	45 min	Visual and semi-quantitative (SUVmax)	
Agrawal et al. [26]	PET/CT (low-dose CT)	^68^Ga-DOTATATE	1.5 MBq/kg	45–60 min	Visual	^18^F-FDG PET/CT
Breer et al. [27]	PET/CT (low-dose CT)	^68^Ga-DOTATATE	58–110 MBq	20 min	Visual and semi-quantitative (SUVmax)	^111^In-octreotide SPECT/CT
Jadhav et al. [28]	PET/CT (low-dose CT)	^68^Ga-DOTATATE	74–111 MBq	60–90 min	Visual	^99m^Tc-octreotide SPECT/CT ^18^F-FDG PET/CT
Clifton-Bligh et al. [29]	PET/CT (low-dose CT)	^68^Ga-DOTATATE	103–226 MBq	45–60 min	Visual	bone scintigraphy ^99m^Tc-sestamibi scintigraphy ^111^In-octreotide scintigraphy ^18^F-FDG PET/CT

Legend: BTV = biologic tumor volume; ^18^F-FDG = fluorine-18 fluorodeoxyglucose; ^68^Ga = gallium-68; MBq = Mega Becquerel; min = minutes; NR = Not reported; PET/CT = Positron Emission Tomography/Computed Tomography; SPECT/CT = Single Photon Emission Computed Tomography/Computed Tomography; SUVmax = maximal Standardized Uptake Value.

**Table 3:** Diagnostic accuracy data of SSTR-PET/CT in the included studies.

Authors	Detection Rate (Patient-Based Analysis)	Site of Culprit Lesion Detected by SSTR-PET/CT					Number of Tumors Detected by SSTR-PET/CT with Histopathology	Histological Type of Culprit Tumors Detected by SSTR-PET/CT
		Cranio-Facial	Trunk	Upper Limbs	Lower Limbs	Metastatic		
John et al. [16]	13/16 (81.3%)	2	1		10		10/13	10 PMT
Pal et al. [17]	20/21 (95.2%)	5	3		12		15/20	11 PMT,2 HP, 1 GCT, 1 HE
Zhang et al. [18] *	37/37 (100%)	5	11	2	19		37/37	35 PMT, 2 SCT
Ding et al. [19]	53/54 (98.1%)	NR	NR	NR	NR	NR	52/53	NR
Paquet et al. [20]	8/11 (72.7%)	1	4		3		8/8	6 PMT, 1 HE, 1 NR
Singh et al. [21]	9/17 (52.9%)	2	2	2	3		7/9	7 PMT
Satyaraddi et al. [22]	8/8 (100%)			1	7		5/8	5 PMT
El-Maouche et al. [23]	6/11 (54.5%)	1	1		3	1	5/6	5 PMT
Bhavani et al. [24]	9/10 (90%)	3	1		5		8/9	6 PMT, 1 HP, 1 SCT
Zhang et al. [25] *	32/32 (100%)	7	5	2	17		32/32	31 PMT, 1 OT
Agrawal et al. [26] *	5/6 (83.3%)	2			3		5/5	2 PMT, 2 HP, 1 OT
Breer et al. [27]	5/5 (100%)	2	1	1	1		5/5	3 PMT, 2 OT
Jadhav et al. [28]	7/7 (100%)	1			6		4/7	NR
Clifton-Bligh et al. [29]	6/6 (100%)			1	5		6/6	6 PMT

Legend: * = excluded from the meta-analysis for possible data overlap; HE = hemangioma; HP = hemangiopericytoma; GCT = giant cell tumor; NR = Not retrieved; OT = odontogenic tumor; PMT = phosphaturic mesenchymal tumor; SCT = spindle cell tumor; SSTR-PET/CT = Somatostatin Receptor Positron Emission Tomography/Computed Tomography.
